# Beyond Conventional Treatment: Herbal Medicine and Nutraceuticals as Complementary Therapies for COPD and Asthma

**DOI:** 10.2174/0118743064454037260510201039

**Published:** 2026-05-14

**Authors:** Ghasaq Al Obaidi, Yusuf Saleeby, Joseph Varon, Natalie Durzynski, Jack Tuszynki, Matthew Halma

**Affiliations:** 1 Internal Medicine, Baghdad Medical City, University of Baghdad, Baghdad, Iraq; 2 Carolina Holistic Medicine, Mount Pleasant, SC, USA; 3 Independent Medical Alliance, Washington, D.C., USA and Dorrington Medical Associates Internal Medicine/Pulmonology, Houston, Texas, USA; 4 Open Source Medicine Foundation, Tallinn, Estonia; 5 Unviersity of Alberta, Edmonton, Alberta, Canada; 6 Politecnico di Torino, Torino, Italy

**Keywords:** Chronic obstructive pulmonary disease, Peak expiratory flow, Postoperative pulmonary function, Complementary therapies, Herbal medicine, Respiratory outcomes, Perioperative care

## Abstract

Chronic obstructive pulmonary disease (COPD) and asthma remain among the most prevalent respiratory disorders worldwide, characterized by chronic inflammation, oxidative stress, and impaired quality of life. Although there has been significant advancement in the pharmacologic therapies, complementary strategies that can potentially target the underlying mechanisms and complement the conventional treatment are growing in interest among numerous patients and providers. This narrative review used systematic search methods in PubMed, Google Scholar, and ScienceDirect to select herbal medicines and nutraceuticals that were studied for COPD and asthma, with a specific selection using objective pulmonary functionality parameters (FEV1, FVC, FEV1/FVC). Analysis of evidence identified multiple interventions with a clinically significant effect, such as *Astragalus membranaceus*, *Rhodiola rosea*, nanocurcumin, Bufei granule, and Wuqinxi breathing exercises, most of which have anti-inflammatory, antioxidant, and immunomodulatory effects. The other agents, including Withania somnifera, Maxingshigan decoction, and L-carnitine, exhibited significant but inconsistent efficacy, whereas compounds like N-acetylcysteine, resveratrol, and cannabis had little effect. Mechanistic research indicates NF-kB, MAPK, Nrf2, and cytokine signaling pathways as typical therapeutic targets. Despite the limitations of methodological heterogeneity, the results indicate the judicious use of the choice of herbal and nutraceutical interventions in comprehensive respiratory care. Such supportive interventions can be patient-centered, enhance medication compliance, and offer an added effect in combination with evidence-based pharmacologic therapies. The quality of therapeutic application of the drug needs to be established through further high-quality therapeutic trials in order to determine the safety, dosing, and long-term outcomes.

## 1 INTRODUCTION

Chronic Obstructive Pulmonary Disease (COPD) and asthma represent two of the most prevalent respiratory disorders globally, affecting over 500 million people worldwide and imposing substantial healthcare and economic burdens [1, 2].

Although these disorders vary in their underlying pathophysiology, they are typified by progressive, mostly permanent airflow restriction and asthma by variable, reversible airflow blockage, which includes chronic inflammation, oxidative stress, and poor life quality [3, 4].

Existing evidence-based interventions have brought about considerable improvement in both diagnoses. The management of COPD is based on bronchodilators, inhaled corticosteroids, pulmonary rehabilitation, and holistic care programs that are effective in reducing the symptoms, preventing exacerbations, and slowing the disease progression [5]. Likewise, the management of asthma with the use of controller therapy, rescue therapy, and avoidance of triggers has allowed the majority of patients to have excellent symptom control and normal functioning [4]. These traditional methods embrace the chronicity of these two diseases and accordingly target the management of symptoms, prevention of exacerbation, and the optimization of quality of life instead of a cure.

Even with these therapeutic advancements, patients and caregivers are turning towards complementary therapies that could offer more value when applied with the traditional therapies. The inflammatory cascades, oxidative damage, immune dysfunction, and airway remo-delling, which are relatively complex and multifactorial, suggest the pathophysiology of underlying mechanisms in chronic respiratory diseases that could be therapeutically synergistic through targeted complementary interventions [6, 7].

### 1.1 Knowledge Gap and the Study's Rationality

Although many single studies have been done to examine herbal medicines and nutraceuticals in respiratory conditions, there is still a wide gap in the synthesis of this evidence. Past reviews have generally used individual interventions or restricted databases, without a systematic analysis of the range of complemen-tary therapies investigated on both COPD and asthma. Moreover, the quality of evidence has not been critically evaluated or clearly given guidance on the interventions that demonstrate the best promise in adjunctive therapies in most of the existing reviews.

#### 1.1.1 Research Objectives and Research Questions

The present narrative review will answer the following specific research questions:

(1) Which herbal medicines and nutraceuticals have shown quantifiable effects on the parameters of pulmonary functions in patients with COPD and asthma?

(2) How do these interventions work to propose their mechanisms of action in managing the pathophysiology of respiratory diseases?

(3) How effective and reliable is the evidence behind these complementary methods?

### 1.2 Primary Objective

To systematically find and analyze herbal medicaments and nutraceutical products that were investigated as complementary therapy to COPD and asthma, synthesizing the existing evidence on their impact on objective pulmonary functioning outcomes.

### 1.3 Secondary Objectives

To outline proposed action mechanisms of promising interventions and the quality of supporting evidence.

The purpose of this review is to bridge the gap between the common practice of traditional medicine and the evidence-based approach to respiratory care and equip clinicians and researchers with a complete resource on the complementary interventions that could be employed to supplement the conventional treatment options used to manage chronic respiratory diseases.

### 1.4 Epidemiology of COPD Incidence

Worldwide prevalence of COPD is estimated at 12.64% for adults 40 years and older as of 2024 [1]. Between 1990 and 2010, COPD prevalence rose 68.9% from 227.3 million cases worldwide to 384 million cases worldwide [2]. Regional prevalence ranges from 15.2% in the Americas to 9.7% in Southeast Asia.

In the USA in 2011, the age-standardized prevalence was reported to be 6.1%, which slightly decreased to 6.0% by 2021 [8]. Another study indicated that the prevalence of COPD among adults aged ≥18 years did not change significantly from 2011 to 2021 [9].

### 1.5 Associated Factors

Demographic factors impact the incidence of COPD, with men historically exhibiting higher rates of COPD [10]. Socioeconomic status also plays a crucial role, with higher prevalence rates in deprived communities [11]. In the United States, non-Hispanic white populations have higher reported rates of COPD compared to other racial and ethnic groups [8].

### 1.6 Trends

COPD trends have been fluctuating in the world. In Norway, a 1995-2008 study reported a rise in the prevalence of COPD in men but not in women, which aligns with the influence of the smoking cessation program and gender-specific smoking trends [12]. Although the smoking rates have decreased in Canada, COPD prevalence rose by 64.8% between 1996 and 2007, with the burden moving increasingly to women [13].

The prevalence rates in the United Kingdom have been stagnant since the mid-1990s among men, and they have been increasing among women until they reached the same prevalence rates between men and women in the late 1990s [10]. Likewise, in Spain, a profound decrease in COPD prevalence was also seen over a decade, with the prevalence decreasing to 4.5% in 2007, and the diagnosis and treatment also improving [14].

In general, although the worldwide prevalence of COPD has been rising, in most high-income countries, age-standardized rates decline or reach a plateau, which is due to the intricate interactions of the aging population, smoking habits, and healthcare advances.

### 1.7 Primary Biomarkers of COPD Diagnosis, Therapy, and Progression

In the assessment of a suspected or known COPD or asthma patient, the examination of the patient usually involves a complete history, physical examination, and diagnostic testing [4, 5]. Clinician initially obtains a detailed medical history with attention given to such symptoms as dyspnea, wheezing, chronic cough, and sputum, exacerbation frequency, and severity. They determine the risk factors such as smoking history, occupational exposures, family history of atopy or respiratory diseases, and environmental triggers. The physical examination is made to determine clinical signs, *i.e*., wheezing, long expiratory sounds, accessory muscle activation, or cyanosis.

To rule out the diagnosis, the clinician might request pulmonary and functional tests (PFTs), especially spirometry to evaluate airflow limitation, reversibility, and lung volumes [15, 16]. In asthma, reversibility of obstruction by bronchodilators is a major diagnostic criterion, unlike COPD, which is marked by persistent airflow limitation [4, 5]. Some other tests, including chest X-rays, computed tomography (CT) scan, or arterial blood gases, can also be conducted to exclude other conditions or assess the severity of the illness. Eosinophil counts in blood or serum IgE levels can both be tested in asthma as part of phenotyping [4].

### 1.8 Pulmonary Function Testing

COPD diagnosis and monitoring are still the gold standards [5, 15]. Key parameters include:

1. FEV1 (Forced Expiratory Volume 1 second)

**Importance:** The maximum volume of air that a person can expel strongly in one second. It is also important in diagnosing COPD [5].

**Role:** Reduced FEV 1 means obstructed airways. The COPD severity is commonly categorized in terms of percentage of predicted FEV1 values [5, 17].

2. FEV 1/ FVC Ratio (Forced Vital Capacity)

**Significance:** The FEV1/FVC ratio can be used to verify the existence of airflow restriction [5, 15].

**Role:** A ratio between post-bronchodilator FEV1 and FVC of less than 0.70 is a diagnostic essential of COPD [5].

3. Peak Expiratory Flow Rate (PEFR)

**Importance:** The maximum rate of expiration [16].

**Role:** Useful in the evaluation of the airway obstruction level and the course of the disease progression [15, 16].

## 2 METHODOLOGY

This narrative review used a systematic approach to identify and review nutraceutical interventions in the management of COPD and asthma. Electronic databases, including PubMed, Google Scholar, and ScienceDirect, were systematically searched using the search terms “herbal medicine COPD review,” “phytotherapy COPD clinical trial,” and “herbal remedies efficacy COPD.” Individual agents were extracted from the reviews shown in **Supplementary Table 1**. References for these agents were extracted from the general reviews.

Although our search strategy was mainly concerned with COPD, we also incorporated evidence relevant to asthma in cases where the agents were found to be effective in both conditions due to the overlapping pathophysiology and similar presentation of the two respiratory conditions.

For each distinct intervention identified, we used specific literature searches with special attention to meta-analyses and systematic reviews that assessed the impact on objective pulmonary functional outcomes in COPD and asthma patients, including FEV1, FVC, and FEV1/FVC ratios. We focused on meta-analyses and systematic reviews to ensure that evidence synthesis relied on the best level of evidence.

This review is a narrative review as opposed to a systematic review due to the heterogeneous nature of complementary medicine research and the quality of studies that are available. Although we used systematic search techniques, the heterogeneous methodologies, outcome measures, and intervention procedures of the studies did not allow formal meta-analysis in the majority of situations.

### 2.1 Interventions that Affect Biomarkers in COPD

The clinical studies and systematic reviews identified 62 distinct herbal, nutraceutical, and complementary interventions. Their effects on pulmonary function were very inconsistent, and only a subset of them reached levels of clinical or definitive significance. A comprehensive summary of these interventions with the effects on parameters of lung function (FEV1, FEV1%, FEV1/FVC), suggested mechanisms of action, and clinical relevance scores is provided in Table **1**. It is worth noting that they included other agents like *Astragalus membranaceus*, *Rhodiola rosea*, nanocurcumin, and Bufei granule, which showed consistent and clinically significant benefits. On the other hand, other agents, including N-acetylcysteine, resveratrol, and cannabis, did not show significance. This section brings out the therapeutic potential of complementary therapies and the limitations of these therapies in COPD and asthma management. The effects of these interventions on the parameters of pulmonary functions (FEV1, FVC, FEV1/FVC), their mechanism of action, and degree of clinical significance are summarized in Table **1**.

Below are the thresholds that were applied to assess the clinical significance of these interventions:

- **FEV1 Minimal Clinically Important Difference (MCID):** >100-140mL (0.1-0.14L)
- **FEV1 Definitive Significance:** >200mL (0.2L) and/or multiple parameters are higher than thresholds
- **FEV1%:** >5-10% for clinical significance
- **FEV1/FVC:** >5-10% for clinical significance

To provide clarity on therapeutic relevance, interventions were stratified according to their impact on pulmonary function parameters. Table **2** emphasizes definitive significant interventions, in which various parameters exceeded minimal clinically significant differences. It is noteworthy that Bufei Granule and *Rhodiola rosea* increased FEV1 and FEV1 percentage and FEV1/FVC simultaneously, and *Astragalus membranaceus* showed dual-parameter importance with great improve-ments in FEV1 and FEV1. Table **3** shows clinically significant interventions which passed single-parameter thresholds, including Withania somnifera and a breathing exercise, Wuqinxi. On the contrary, Table **4** provides a summary of those agents that did not achieve clinical thresholds, such as N-acetylcysteine, resveratrol, and cannabis. Lastly, Table **5** provides a priority ranking framework of integrative COPD management, placing therapeutic options within a scale of strongest evidence (Multi-parameter interventions) to limited evidence availability. Collectively, these tables can be used to generalize the scope of herbal, nutraceutical, and complementary therapies considered, which offers a systematic evidence base upon which one can base the application of these therapies in clinical practice.

### 2.2 Clinical Significant Interventions and Mechanisms of Action

#### 2.2.1 Astragalus Membranaceus

*Astragalus membranaceus* has several active components that have demonstrated potential in the management of Chronic Obstructive Pulmonary Disease (COPD). Its active constituents are largely astragaloside IV, astragalus polysaccharides (APS), flavonoids (calycosin and formononetin), as well as saponins. One of its main constituents, astragaloside IV, showed several positive effects in the treatment of COPD. It is capable of reducing oxidative stress, suppressing the presence of reactive oxygen species (ROS), and balancing the Nrf2/HO-1 pathway [70]. This compound also suppresses the TGF-β1/Smad signaling pathway that suppresses the deposition of the extracellular matrix and airway remodeling [71]. Moreover, astragaloside IV has been found to exhibit anti-inflammatory effects by reducing the production of pro-inflammatory cytokines like IL-8 and elevating the production of anti-inflammatory cytokines like IL-10 [70].

APS are very important in the treatment of COPD symptoms. They have been detected to reduce the levels of hydroxyproline in pulmonary tissues and repress MMP-9 protein synthesis in COPD rats, to counteract the formation and degradation of extracellular matrix and alleviate ineffective airway restructuring [7]. APS also exhibits antioxidant properties, enhancing the body's antioxidant capacity by increasing levels of antioxidant markers such as Superoxide Dismutase (SOD) and Glutathione Peroxidase (GSH-Px) [70].

The anti-inflammatory effect of Astragalus membranaceus is due to flavonoids, especially calycosin and formononetin. Cell experiments have demonstrated a decrease in IL-8 and Matrix Metalloproteinases (MMP)-9 secretions with these compounds showing possible therapeutic effects on COPD [70]. Formononetin has also been reported to suppress the release of arachidonic acid in stimulated macrophages, which may also be added to the total anti-inflammatory action of Astragalus extracts [72].

The synergistic effect of these active ingredients in *Astragalus membranaceus* is aimed at treating various COPD pathology aspects. Their combined effect increases the production of nitric oxide, helps in the improvement of the endothelial functionality, antioxidant protection, and in the control of the inflammatory reactions [73]. This is a multi-targeted strategy that can be used to manage the symptoms, enhance lung functioning, and even decelerate the progression of the disease in COPD patients.

#### 2.2.2 Maxingshigan Decoction

Maxingshigan decoction (MSD) is a traditional Chinese medicine formula that is employed in the treatment of respiratory diseases such as COPD. The decoction is composed of four main herbs: *Ephedra sinica Stapf* (Mahuang), *Prunus armeniaca* L. (Kuxingren), Gypsum fibrosum (Shigao), and *Glycyrrhiza uralensis Fisch*. (Gancao) [74, 75].

Each of these herbs contains active ingredients that contribute to the overall therapeutic effect of MSD in COPD treatment. Ephedra sinica contains ephedrine and pseudoephedrine as its major alkaloids. These compounds act as non-selective sympathomimetic agents with both alpha and beta adrenergic activities [76]. Ephedrine has a relatively long-lasting antispasmodic effect on bronchial smooth muscle, which helps in relieving bronchospasm associated with COPD. It also stimulates the central nervous system, leading to bronchodilation and improved respiratory function [76].

*Prunus armeniaca* L. contains amygdalin as its main active ingredient. When metabolized, amygdalin produces hydrocyanic acid in the body, which has an inhibitory effect on the respiratory center [4]. This action contributes to the antitussive properties of MSD. Moreover, *Prunus* *armeniaca* L. has demonstrated anti-inflammatory, anti-oxidant, and immunomodulatory properties, which can be used to control COPD symptoms [6].

Gypsum fibrosum, primarily composed of calcium sulfate, has been shown to have an inhibitory effect on white blood cell pyrogenic fever and can improve cellular immune function [74, 77]. It may also play a role in regulating intracellular calcium levels, which could influence smooth muscle contraction in the airways [77].

*Glycyrrhiza uralensis fisch.* contains glycyrrhizic acid as its main active compound. Glycyrrhizic acid has broad-spectrum antiviral activity and anti-inflammatory effects [78]. It suppresses TNF-alpha and caspase-3, inhibits the translocation of NF-κB into the nuclei, and conjugates free radicals [78].

These activities help to decrease respiratory inflammation in the airways, one of the main characteristics of COPD. These combined herbs in MSD act synergistically to give a multifactorial treatment of COPD. The decoction has also been reported to have a beneficial effect in inhibiting neutrophilic airway inflammation by suppressing the expression of inflammatory cytokines and CXCL-2 by inhibiting the IL-17/STAT3 pathway [79].

The combination of the active ingredients in Maxingshigan decoction produces bronchodilation, anti-inflammatory, immunomodulatory effects, and antitussive effects, all of which are useful in the treatment of COPD symptoms and progression.

#### 2.2.3 Ginseng

Panax ginseng has several active components that are potentially useful in the treatment of chronic obstructive pulmonary disease (COPD). The main active constituents of ginseng are ginsenosides, especially ginsenosides Rb1, Rd, Re, Rg1, Rg2, Rg3, Rh1, and Rh2 [80].

These ginsenosides have various modes of action in COPD therapy. Ginsenosides exhibit strong anti-inflammatory properties through the inhibition of pro-inflammatory cytokines like TNF-α, IL-6, and IL-8 [80].

They also regulate inflammatory signatures by preventing the induction and translocation of NF-kB, the phosphorylation of kinases, such as MAPK and ERK1/2 [81]. Moreover, ginsenosides inhibit reactive oxygen species (ROS) and protease activities such as MMP-9, which take part in COPD pathogenesis [80, 81].

Ginseng and its compounds are also of great antioxidant value. They prevent oxidative stress through the enhancement of antioxidant enzyme activity and decreasing the generation of oxidants [82]. Such anti-oxidant action is useful in vascular endothelium protection and could be one of the factors behind the overall cardio-vascular effects found in COPD patients [83]. Moreover, it has been demonstrated that ginsenosides can increase the production of nitric oxide and improve the endothelial activity [83]. Such a vasodilatory effect can be useful in enhancing lung performance in patients with COPD. Research has also established that ginseng supplementation is able to enhance the forced expiratory volume at one second (FEV1) among patients with COPD [82].

The immunomodulatory effect of ginseng is also available, and this can be useful in the treatment of COPD. It has also proven to activate the immune system, and this may help the body to combat respiratory infections that may worsen COPD symptoms [80].

Although these results are promising, it is worth mentioning that further studies are required to completely prove the effectiveness and the effective dosage of ginseng in the treatment of COPD. The multifaceted interaction of numerous proactive substances in ginseng and their diverse action mechanisms renders it a fasci-nating topic of research in COPD management [82, 83].

#### 2.2.4 Rhodiola rosea L. (RRL)

*Rhodiola rosea* L. (RRL) has demonstrated potential benefits in the treatment of COPD in several ways. The potent antioxidant, anti-inflammatory, and antifibrotic effects of rosin, as well as salidroside, tyrosol, rosavin, rosarin, and rosin, are demonstrated as the active ingredients of RRL.

These substances have a synergistic effect in the reduction of the symptoms and progression of COPD. RRL has been shown to modulate lung function parameters in COPD patients, including forced expiratory volume in 1 second (FEV1), FEV1/FVC ratio, and partial pressure of oxygen in arterial blood (PaO_2_) significantly [32, 84]. It also decreases inflammatory, *e.g*., C-reactive protein (CRP), and antioxidant capacity, *i.e*., superoxide dismutase (SOD) and glutathione (GSH) levels, [32, 84].

The mechanism of action of RWL has been described to control the critical pathways of inflammation, such as suppression of NF-kB and activation of Nrf2 and HO-1[85, 86], and has been shown to prevent COPD caused by cigarette smoking and lipopolysaccharide by suppressing the ERK1/2/Smad3 signaling pathway and apoptosis [86]. Moreover, it has been indicated to reduce the adhesion of inflammatory cells and enhance the pathology of the lung tissue in COPD models [86].

#### 2.2.5 Curcumin

The active constituent of turmeric, *Curcuma longa*, is curcumin, which has demonstrated possible benefits in the management of COPD with various mechanisms of action. Carcinogenic and other curcuminoids are the key active components of curcumin, which give it its therapeutic effects in COPD. They are potent anti-inflammatories and antioxidants that can be used in the management of COPD symptoms and progression [7, 87].

The anti-inflammatory actions of curcumin are facilitated by blocking the activities of pro-inflammatory cytokines like TNF-α, IL-6, and IL-8. It also regulates inflammatory pathways through the inhibition of NF-kB transcription factor induction and translocation and the inhibition of kinase phosphorylation, including MAPK and ERK1/2 [87, 88]. Moreover, curcumin inhibits the generation of reactive oxygen species (ROS) and proteases such as MMP-9 that are involved in the pathogenesis of COPD [7, 89]. The antioxidant effect of curcumin is essential in the prevention of oxidative stress, which is an important contributor to COPD formation. Curcumin enhances antioxidant enzyme activity, including glutathione peroxidase (GPx), superoxide dismutase (SOD), and catalase (CAT), and total antioxidant capacity [89, 90]. This antioxidant effect has been found to protect the vascular endothelium and could potentially benefit the general lung health in COPD patients [89, 90].

Curcumin also demonstrates airway inflammatory effects, inhibits emphysema, and prevents ischemic complications related to COPD [7, 88] and improves nitric oxide generation and endothelium function, which has the potential to benefit the lung functioning of COPD patients [7].

#### 2.2.6 Withania Somnifera

Root extract of *Withania somnifera* (WS) is rich in several phytochemicals with inhibitory effect on the major proteins that play a role in COPD pathogenesis. *In silico* research has indicated that withanolides in WS roots exhibit a significant inhibitory effect on angiotensin-converting enzyme 2 (ACE-2), myeloperoxidase (MPO), and interleukin-6 (IL-6) [42]. Moreover, WS was also demonstrated to modulate a variety of cytokines, stimulate T-cell proliferation, and stimulate the activity of macrophages, which promotes its immunomodulatory properties [91]. This immunomodulation assists in the regulation of the humoral and cellular responses of the adaptive immune system, which is very helpful in dealing with the symptoms of COPD. WS components, including withaferin A, have been identified as one of the active constituents and inhibitors of nuclear factor kappa-B activation, which is a major controller of inflammatory reactions [92].

#### 2.2.7 Bufei Granule

Bufei Jianpi granule (BJG) is an ancient Chinese medicine compound that is applied in the treatment of COPD. BJG has been shown to reduce inflammatory factors such as IL-6, IL-8, TNF-α, PGE2, MMP-9, and NO in serum and bronchoalveolar lavage fluid (BALF) of rats with COPD [93]. The formula is also indicated to have antioxidant properties, which also aid in better functioning of the lungs among COPD patients [57].

A few of the active components identified are ginsenoside Rh1, paeonol, icariin, nobiletin, and astragaloside A [57]. These agents are proven to be therapeutic agents in enhancing COPD conditions by acting on EGFR, ERK1, and PAI-1 targets [57].

#### 2.2.8 Wuqinxi

The traditional Chinese mind-body exercise, which is called Wuqinxi, has demonstrated encouraging results in the management of COPD. Wuqinxi acts on COPD in a multifaceted mechanism. Similar to other types of Qigong, Wuqinxi uses diaphragmatic breathing and pursed-lip breathing, which may assist a COPD patient to reduce their breathing rate and avoid premature airway closure due to rapid airflow [94].

This breathing pattern improvement increases the functionality of the auxiliary breathing muscles and the flexibility, coordination, and control ability of the neuromuscular limbs, ultimately leading to an increase in the exercise capacity of COPD patients [94]. Moreover, Wuqinxi has been demonstrated to enhance the quality of life of COPD patients, based on the Clinical COPD Questionnaire (CCQ). The meta-analysis provided an SMD of 1.23 (95% CI 0.31 to 2.14, *p* = 0.01) on CCQ scores [95].

It is worth pointing out that these findings are encouraging, but additional clinical trials of high quality are required to comprehensively determine the effectiveness and most effective use of Wuqinxi in the treatment of COPD [94]. Nevertheless, due to the lack of any adverse effects, clinicians may consider the inclusion of Wuqinxi exercise in the rehabilitation procedure of COPD patients (30-45min exercise per day) [56].

#### 2.2.9 Xiaoqinglong

Xiaoqinglong decoction (XQLD) is a Chinese herbal formula that may be used to treat COPD. Paeonol, glycyrrhizin, and geranidin are the active ingredients in XQLD that cause its therapeutic action on COPD [96]. XQLD exhibits multiple mechanisms of action in treating COPD. It has been shown to inhibit the progression of COPD by attenuating the autophagy process [97]. The formula can improve airway hyperreactivity and airway reconstruction, as well as alleviate airway inflammation in COPD [96].

XQLD has been found to reduce the production of pro-inflammatory cytokines such as IL-4 and IL-5 in airway inflammatory models [96]. It also increases FoxP3+ and CD4+ T cells, which play a role in immune regulation [96]. The antioxidant properties of XQLD contribute to its therapeutic effects in COPD. It helps rectify the imbalance of oxidation/anti-oxidation and alleviates inflammatory reactions [96].

XQLD has been identified to be capable of increasing rates of apoptosis, up-regulating the LC3 II/LC3 I ratio, and down-regulating p62 levels in COPD models [97]. It also regulates the NF-kB and g-GCS in rats with COPD [96].

### 2.3 Yuxingcao or Herba Houtuyniae

Herba Houttuyniae or Yuxingcao (*Houttuynia cordata*) is a group of active constituents that have demonstrated promise in the treatment of COPD, and is a plant with multiple active constituents. The principal active ingredients are flavonoids (quercitrin, hyperoside, rutin), alkaloids, and volatile oils [97-99]. The herb's active components modulate the PI3K/AKT, MAPK, and TNF signaling pathways, which are crucial in the progression of pulmonary fibrosis, a common complication in advanced COPD [100]. Polysaccharides extracted from *Houttuynia cordata* have demonstrated immunomodulatory effects, inhibiting the expression of inflammatory mediators and reducing nitric oxide production in a dose-dependent manner [101].

## 3 DISCUSSION

Despite being a simple and readily available measure of pulmonary mechanics, the clinical applicability of peak expiratory flow (PEF) ultimately remains unknown until it can be determined whether the change observed after surgery can be translated into meaningful patient outcomes. The existing perioperative and respiratory literature has shown that postoperative impairments in lung functioning are linked to further dangers of atelectasis, pneumonia, requirement of respiratory physiotherapy, and extended hospital stay, especially in the setting of abdominal and thoracic surgery [102, 103]. Nonetheless, the literature meta-analyzed in the current review mostly presented the physiological outcomes of PEF or spirometric modifications, without always connecting these sufficiently with subsequent clinical endpoints. Therefore, although decreases in PEF are valid and reliable indicators of impaired respiratory mechanics due to the consequences of surgical stress, anesthesia, and splinting during pain, their prognostic utility is not sufficiently established in the existing body of evidence.

Notably, a significant percentage of interventions considered were unable to prove a clinically meaningful benefit. The effects of widely available compounds, including N-acetylcysteine, resveratrol, and cannabis, had a slight or variable impact on the parameters of pulmonary functions, and their reported effects were less than the recognized limits of clinical significance [104-106]. The evidence base of most non-traditional Chinese medicine interventions is notably minimal and is often typified by small sample sizes, non-homogeneous dosing schedules, and reporting inconsistency of outcomes. Such null results can be clinically interpreted and serve as an indication of the necessity of interpreting small physiological variations without necessarily meriting any reproducible benefit.

## 4 LIMITATIONS AND FUTURE DIRECTIONS

There are some limitations that should be considered. To begin with, the existing research mostly presents the parameters of pulmonary functions, such as PEF and the spirometric indices, in an unsystematic association with clinically significant postoperative events, including pulmonary complications, length of hospital stay, or readmission. Second, inconsistent reporting of early postoperative measurements (which could be a more accurate measure of respiratory impairment nadir) was observed across the studies. Third, much of the evidence relies on single-center studies, which have small samples and are not generalizable. Finally, no tested PEF cutoffs that can be depended on to outline pulmonary complications following surgery have been tested, thus necessitating a prospective research involving serial pulmonary monitoring of functions as well as the traditional clinical outcomes [107].

This review should be interpreted within the scope of the large methodological heterogeneity among studies included. There is a lot of variability in the study design, sample size, composition of intervention, dosing schedule, period of treatment, and outcome measurement, making direct comparability across the trials challenging. The quality of studies is also characterized by a great variety, with a lot of interventions being backed by small, single-center studies. Due to this heterogeneity and non-aggregated reporting of the results, formal meta-analysis was not possible, and the evidence was thus synthesized through a narrative review method, which is in line with the existing recommendations regarding the synthesis of the evidence in heterogeneous bodies of clinical work [108].

The interpretation of evidence in complementary and nutraceutical studies is also made complex by well-known methodological problems in the biomedical literature, such as immortal time bias, selective publication of results, and interpretive spin [109, 110]. These problems are especially applicable in areas where the study designs are heterogeneous, the standardization of the formulation of the variables is inconsistent, and the selection of outcomes is also unstable, as is the case in the research of herbal medicines. Also, inconsistency in reporting quality and frequent retractions in the wider biomedical literature reflect the need to exercise caution when dealing with relatively small effect sizes, or in a study design that does not provide the appropriate level of control. These limitations in methodology would be useful in contextualizing the reported benefits and also in directing the design of more transparent, reproducible, and clinically informative trials in the future.

Future studies need to go beyond single physiological endpoints to more integrative analytical methods that are able to deal with the complex and heterogeneous data. The future of artificial intelligence, especially large machine-learning models, will provide potentially helpful tools in discerning response trends, optimizing the choice of intervention, and enhancing the process of evidence synthesis in a wide range of trials of herbal medicine [111, 112]. In particular settings where the intervention formulations, dosing strategies, and outcome measures differ significantly, such techniques would be particularly helpful in enhancing transparency, interpretability, and reproducibility in complementary respiratory medicine studies.

## CONCLUSION

This narrative review represents a synthesis of existing evidence on the herbal medicines and nutraceuticals that have been tested as complementary therapies in COPD and asthma, as well as their effect on pulmonary functions. Although the interventions chosen demonstrate reproducible changes in physiological parameters, the evidence base in general is limited due to methodological heterogeneity, small samples, and inconsistent reporting of outcomes. Subsequently, the results of the observed outcomes are to be viewed with caution and cannot yet be applied to definite clinical guidelines [113].

The results endorse the notion that complementary therapies can be used as a complement to, and not a replacement for, the usual pharmacologic treatment until additional studies confirm them. The future studies ought to focus on rigorously designed randomized controlled trials whose interventions are standardized, with clinically relevant endpoints, and the long-term safety analysis to achieve clarity on the therapeutic relevance. These are necessary endeavors towards evaluating whether physiological enhancements reported in the available literature translate into long-term clinical outcomes in integrated, evidence-based respiratory care.

## Figures and Tables

**Table. 1:** Summary of 62 herbal, nutraceutical, and complementary interventions evaluated in COPD and asthma with reported effects on pulmonary function parameters (FEV_1_, FVC, FEV_1_/FVC), the mechanism of action for each intervention and level of clinical significance.

Intervention	FEV1 (L) [95% CI]	FEV1% [95% CI]	FEV1/FVC [95% CI]	Other Parameters	Active Components/Mechanisms	Clinical Significance Rating	Reference
*Astragalus membranaceus* (with ambroxol HCL)	0.30 [0.18, 0.42]	16.18 [12.60, 19.76]	2.51 [-0.05, 5.06]	-	Astragaloside IV, polysaccharides, flavonoids; alleviates oxidative stress, inhibits airway remodeling, reduces IL-8, increases IL-10	Definitive (FEV1 >0.2L + FEV1% >10%)	[18]
*Astragalus membranaceus* - alternative study	0.30 [0.24, 0.36]	-	2.80 [2.03, 3.57]	-	Astragaloside IV, polysaccharides, flavonoids; alleviates oxidative stress, inhibits airway remodeling, reduces IL-8, increases IL-10	Definitive (FEV1 >0.2L)	[19]
*Glycyrrhiza glabra* (Licorice)	n.s.	-	-	-	Glycyrrhizic acid: antiviral, anti-inflammatory effects	Not Significant	[20]
Maxingshigan decoction	-	13.8 [8.2, 19.4]	-	-	Ephedra sinica (ephedrine, pseudoephedrine), Prunus armeniaca L. (amygdalin), Gypsum fibrosum (calcium sulfate), Glycyrrhiza uralensis; reduces neutrophilic airway inflammation via IL-17/STAT3 pathway	Clinically Significant (FEV1% >10%)	[21]
Dang Shen	0.22 [0.13, 0.31]	-	-	-	-	Definitive (FEV1 >0.2L)	[22]
Panax ginseng	n.s.	-	n.s.	-	Ginsenosides; inhibits TNF-α, IL-6, IL-8; modulates NF-κB, MAPK, ERK1/2 pathways	Not Significant	[23]
Ginseng - alternative study	0.30 [0.02, 0.58]	9.43 [3.64, 15.21]	-	-	Ginsenosides; inhibits TNF-α, IL-6, IL-8; modulates NF-κB, MAPK, ERK1/2 pathways	Definitive (FEV1 >0.2L + FEV1% >5%)	[24]
*Zingiber officinale* (Red Ginger)	-	-	2.08 [1.40, 2.76]	-	-	Not Significant (FEV1/FVC <5%)	[25]
*Ephedra sinica* and rhubarb	-	4.72 [1.19, 8.25] (*vs.* Western Medicine)	n.s. (but reported as significant)	-	-	Not Significant (FEV1% <5%)	[26]
*Glycyrrhiza uralensis*	-	22 [95% CI not reported] (asthmatic patients)	-	-	-	Clinically Significant (FEV1% >10%)	[27]
San-Huang Gu-Ben Zhi-Ke	n.s.	n.s.	n.s.	-	-	Not Significant	[28]
Huangqi	0.19 [0.10, 0.28]	Significant improvement (value not accessible)	Significant improvement (value not accessible)	-	-	Clinically Significant (FEV1 >0.14L MCID)	[29]
Huangqi - alternative study	Significant improvement (value not accessible)	-	-	-	-	Clinically Significant (reported significant)	[30]
*Nigella* (Thymoquinone)	-	-	n.s.	-	-	Not Significant	[31]
*Rhodiola rosea*	0.25 [0.21, 0.29]	9.28 [4.41, 14.15]	5.04 [2.64, 7.43]	-	Salidroside, tyrosol, rosavin, rosarin; antioxidant, anti-inflammatory, antifibrotic; modulates NF-κB, Nrf2, HO-1 pathways	Definitive (FEV1 >0.2L + FEV1% >5% + FEV1/FVC >5%)	[32]
Modified dachengqi decoction	-	5.3 [1.48, 9.12]	1.55 [0.23, 2.87]	-	-	Clinically Significant (FEV1% >5%)	[33]
Crocin	0.15 [95% CI not available]	-	n.s.	-	-	Clinically Significant (FEV1 >0.14L MCID)	[34]
*Zataria multiflora*	1	-	-	-	-	Definitive (FEV1 >0.2L)	[35]
*Nepeta bracteata*	n.s.	-	n.s.	-	-	Not Significant	[36]
Nanocurcumin	-	13.03 [7.6, 18.47]	11.62 [7.3, 15.93]	-	Curcuminoids; inhibits TNF-α, IL-6, IL-8; modulates NF-κB, MAPK, ERK1/2; increases GPx, SOD, CAT	Clinically Significant (FEV1% >10% + FEV1/FVC >10%)	[37]
Ivy leaf extract	-	n.s.	n.s.	-	-	Not Significant	[38]
*Hedera helix*	1	-	-	-	-	Definitive (FEV1 >0.2L)	[39]
*Solanum trilobatum*	1	-	-	-	-	Definitive (FEV1 >0.2L)	[40]
*Solanum xanthocarpum*	1	-	-	-	-	Definitive (FEV1 >0.2L)	[40]
Inflaminat (combination: *Sambucus nigra, Viola tricolor, Calendula officinalis*)	-	n.s.	n.s.	-	-	Not Significant	[41]
*Withania somnifera*	-	5.43 [2.00, 8.90]	4.93 [2.53, 7.33]	-	Withanolides; inhibit ACE-2, MPO, and IL-6; modulate cytokines, enhance T-cell proliferation and macrophage functions	Clinically Significant (FEV1% >5%)	[42]
Chinese herbal medicine (General)	0.12 [0.08, 0.16] (MD)	-	-	-	-	Clinically Significant (FEV1 within MCID range)	[43]
Buzhong Yiqi Tang	-	6.66 [5.79, 7.54]	-	-	-	Clinically Significant (FEV1% >5%)	[44]
Cordyceps	-	n.s.	7.2 [2.56, 11.84]	-	-	Clinically Significant (FEV1/FVC >5%)	[45]
Shufeng Jiedu	-	-	4.83 [2.56, 7.10]	-	-	Not Significant (FEV1/FVC <5%)	[46]
Weijing decoction - Study 1	0.23 [0.16, 0.29]	-	-	-	-	Definitive (FEV1 >0.2L)	[47]
Weijing decoction - Study 2	0.25 [0.19, 0.30]	-	-	-	-	Definitive (FEV1 >0.2L)	[48]
Herbal medicine (General)	0.14 [0.03, 0.24]	-	-	-	-	Clinically Significant (FEV1 = 0.14L MCID threshold)	[49]
Qingqihuatan decoction	0.34 [0.10, 0.58]	-	-	-	-	Definitive (FEV1 >0.2L)	[50]
Qingjin Huatan	0.29 [0.08, 0.51]	-	-	-	-	Definitive (FEV1 >0.2L)	[50]
Qingkailing	0.20 [0.06, 0.35]	-	-	-	-	Definitive (FEV1 = 0.2L threshold)	[51]
Reduning - Study 1	0.24 [0.08, 0.40]	-	-	-	-	Definitive (FEV1 >0.2L)	[51]
Tanreqing	0.24 [0.19, 0.29]	-	-	-	-	Definitive (FEV1 >0.2L)	[51]
Reduning - Study 2	0.26 [0.20, 0.32]	-	-	-	-	Definitive (FEV1 >0.2L)	[51]
Yuxingcao (herba houttuyniae)	0.73 [0.06, 1.41]	-	-	-	Flavonoids (quercitrin, hyperoside, rutin), alkaloids, volatile oils; modulates PI3K/AKT, MAPK, TNF pathways	Definitive (FEV1 >0.2L)	[51]
Chinese oral herbal paste	-	3.09 [1.49, 4.69]	-	-	-	Not Significant (FEV1% <5%)	[52]
N-acetyl cysteine (NAC)	n.s.	-	-	FEV1 improvement: 0.031 [0.025, 0.036] L	Antioxidant properties	Not Significant (FEV1 <0.1L MCID)	[53]
Xiaoqinglong decoction	0.37 [0.27, 0.46]	5.11 [4.21, 6.00]	-	-	Paeonol, glycyrrhizin, geranidin; inhibits autophagy, reduces IL-4, IL-5; increases FoxP3+, CD4+ T cells	Definitive (FEV1 >0.2L + FEV1% >5%)	[54]
Xuefu Zhuyu decoction	0.16 [0.06, 0.26]	-	-	-	-	Clinically Significant (FEV1 >0.14L MCID)	[55]
Baduanjin (Exercise)	-	-	3.45 [0.49, 6.44]	-	Mind-body exercise with breathing techniques	Not Significant (FEV1/FVC <5%)	[56]
Liuzijue (Exercise)	-	-	4.43 [1.12, 7.70]	-	Traditional breathing exercise	Not Significant (FEV1/FVC <5%)	[56]
Taijiquan (Exercise)	-	-	4.05 [0.71, 7.34]	-	Mind-body exercise	Not Significant (FEV1/FVC <5%)	[56]
Wuqinxi (Exercise)	-	-	8.62 [4.46, 13.04]	-	Traditional Chinese mind-body exercise incorporates diaphragmatic and pursed-lip breathing; improves neuromuscular flexibility and control	Clinically Significant (FEV1/FVC >5%)	[56]
Bufei granule	0.37 [0.34, 0.40]	8.05 [3.86, 12.23]	7.53 [4.42, 10.63]	-	Ginsenoside Rh1, paeonol, icariin, nobiletin, astragaloside A; lowers IL-6, IL-8, TNF-α, PGE2, MMP-9, NO	Definitive (FEV1 >0.2L + FEV1% >5% + FEV1/FVC >5%)	[57]
Shenling baizhu san	n.s.	n.s.	6.83 [5.40, 8.27]	-	-	Clinically Significant (FEV1/FVC >5%)	[58]
Yiqibushenhuoxue	-	n.s.	n.s.	-	-	Not Significant	[59]
Compound Honey Syrup (CHS)	0.25 [0.04, 0.46]	4.66 [0.59, 8.73]	-	PEFR: 5.37 [1.36, 9.38]	-	Definitive (FEV1 >0.2L)	[60]
Tualang honey	n.s.	-	n.s.	-	-	Not Significant	[61]
Jeeva^®^ (Plant-based Nutraceutical) - Study 1	n.s. 0.26 [-0.12, 0.64] (*vs.* baseline)	-	n.s. 1.73 [-3.74, 7.21] (*vs.* baseline)	-	-	Not Significant	[62]
Jeeva^®^ (Plant-based Nutraceutical) - Study 2	0.16 [-0.25, 0.57]	-	n.s. 0.67 [-1.44, 3.77]	-	-	Clinically Significant (FEV1 >0.14L MCID)	[63]
Cannabis	0.013 [0.006, 0.020] per year of daily joint use	-	-	FVC by 20mL/joint-year (95% CI, 12 to 27; *p* < .001),	-	Not Significant (FEV1 <0.1L MCID)	[64]
Glutamine and glycine	0.026 [0.017, 0.051] per SD difference in metabolite levels	-	-	-	Metabolic association with lung function	Not Significant (FEV1 <0.1L MCID)	[65]
Hydrocinnamate	0.026 [0.017, 0.051] per SD difference in metabolite levels	-	-	-	Metabolic association: phenolic compound with antioxidant properties	Not Significant (FEV1 <0.1L MCID)	[65]
Resveratrol	n.s.	-	-	-	Polyphenolic compound; improves heart function by moderating inflammatory processes in systolic heart failure	Not Significant	[66]
L-carnitine	Correlation with plasma total carnitine (r=0.77)	-	-	-	Amino acid derivative; involved in fatty acid metabolism; correlates with FEV1 levels in COPD patients	Clinically Significant (Strong positive correlation)	[67]
Sauna	-	-	-	PEFR: 0.48 ± 0.79 L/s (*vs.* control: -0.11 ± 0.72 L/s)	Heat therapy may improve cardiovascular and respiratory function	Clinically Significant (Improved PEFR *vs.* control)	[68]
Umckaloabo	n.s.	-	-	-	Pelargonium sidoides extract; traditional respiratory remedy	Not Significant	[69]

**Note:** n.s: not significant

**Table 2:** Definitive significance interventions (FEV1 >0.2L and/or multiple parameters are higher than thresholds).

Rank 1 - Triple Parameter Significance				
Intervention	FEV1 (L)	FEV1%	FEV1/FVC	Clinical Rating
Bufei Granule	0.37	8.05%	7.53%	Definitive - All 3 parameters
*Rhodiola rosea*	0.25	9.28%	5.04%	Definitive - All 3 parameters
Rank 2 - Dual Parameter Significance				
Intervention	FEV1 (L)	FEV1%	FEV1/FVC	Clinical Rating
*Astragalus membranaceus* (with ambroxol HCL)	0.30	16.18%	-	Definitive - FEV1 + FEV1%
Xiaoqinglong decoction	0.37	5.11%	-	Definitive - FEV1 + FEV1%
Ginseng	0.30	9.43%	-	Definitive - FEV1 + FEV1%
Nanocurcumin	-	13.03%	11.62%	Definitive – FEV% + FEV1/FVC
Rank 3 - Single Parameter Significance (FEV1 >0.2L)				
Intervention	FEV1 (L)	Clinical Rating		
Yuxingcao	0.73	Definitive		
Qingqihuatan decoction	0.34	Definitive		
Qingjin Huatan	0.29	Definitive		
Reduning	0.26 / 0.24 (two studies)	Definitive		
Weijing Decoction	0.25 / 0.23 (two studies)	Definitive		
Compound honey syrup	0.25	Definitive		
Tanreqing	0.24	Definitive		
Dang Shen	0.22	Definitive		
Qingkailing	0.20	Definitive		
*Astragalus membranaceus* (alternative study)	0.30	Definitive		
*Zataria multiflora*	1.0	Definitive		
*Hedera helix*	1.0	Definitive		
*Solanum trilobatum*	1.0	Definitive		
*Solanum xanthocarpum*	1.0	Definitive		

**Table 3:** Clinically significant interventions (meeting single parameter thresholds).

Intervention			FEV1%	Clinical Rating
*Glycyrrhiza uralensis*			22% in asthmatic patients	Clinically significant
Maxingshigan decoction			13.8%	Clinically significant
Buzhong Yiqi Tang			6.66%	Clinically significant
*Withania somnifera*			5.43%	Clinically significant
Modified dachengqi decoction			5.3%	Clinically significant
**FEV1/FVC Focused (>5%)**				
**Intervention**			**FEV1/FVC**	**Clinical Rating**
Wuqinxi			8.62%	Clinically significant
Cordyceps			7.2%	Clinically significant
Shenling Baizhu San			6.83%	Clinically significant
**FEV1 MCID Range (0.1-0.14L)**				
**Intervention**			**FEV1 (L)**	**Clinical Rating**
Huangqi			0.19	Clinically significant
Xuefu Zhuyu decoction			0.16	Clinically significant
Jeeva^®^ (Study 2)			0.16	Clinically significant
Crocin			0.15	Clinically significant
Herbal medicine			0.14	Clinically significant
Chinese herbal medicine			0.12	Clinically significant
**Other Parameters**				
**Intervention**	**Parameter**		**Clinical Rating**	
L-carnitine	Correlation with FEV1 (r=0.77)		Clinically significant	
Sauna	PEFR improvement *vs.* control			

**Table 4:** Not clinically significant interventions.

Intervention	Result	Reason
N-acetyl cysteine (NAC)	0.031L, n.s.	Below the MCID threshold
Resveratrol	n.s.	Not significant for FEV1
Umckaloabo	n.s.	Not significant for FEV1
Hydrocinnamate	0.026L per SD	Below the MCID threshold
Cannabis	0.013L per year	Very small effects
Glutamine and glycine	0.026L per SD	Very small effects
Most exercise interventions	Various	Except Wuqinxi
20+ other interventions	n.s.	Not significant results

**Note:** n.s: not significant

**Table 5:** Priority ranking for integrative COPD treatment.

Tier	Category	Interventions	Rationale
Tier 1	Definitive multi-parameter	• *Bufei Granule* • *Rhodiola rosea*	Triple parameter excellence
Tier 2	Definitive dual-parameter	• *Astragalus membranaceus* (with ambroxol HCL) • Xiaoqinglong decoction. Ginseng. Nanocurcumin	Dual parameter significance
Tier 3	Definitive single-parameter	• Yuxingcao • Qingqihuatan decoction • High-FEV1 herbs • Astragalus (alternative study)	FEV1 >0.2L
Tier 4	Clinically significant	• Maxingshigan decoction • Wuqinxi • L-carnitine (correlation) • Sauna (PEFR)	Single parameter thresholds
Tier 5	Limited evidence	• NAC • Resveratrol • Umckaloabo • Hydrocinnamate	Below significance thresholds

## LIST OF ABBREVIATIONS

CT: Computed Tomography
COPD: Chronic Obstructive Pulmonary Disease
PEFR: Peak Expiratory Flow Rate
MCID: Minimal Clinically Important Difference
