# Supplementary material for: Beyond Conventional Treatment: Herbal Medicine and Nutraceuticals as Complementary Therapies for COPD and Asthma
Source: Open Respir Med J. 2026 May 14;20:e18743064454037. doi: 10.2174/0118743064454037260510201039 (PMC13458475; doi:10.2174/0118743064454037260510201039)
Supplement: Supplementary file 1 [file TORMJ-20-E18743064454037_SD1.pdf]

The Open Respiratory Medicine Journal
ISSN: 1874-3064
DOI: 10.2174/0118743064454037260510201039, 2026, 20, e18743064454037
1
SUPPLEMENTARY MATERIAL
OPEN ACCESS

Beyond Conventional Treatment: Herbal Medicine and Nutraceuticals as Complementary Therapies for COPD and Asthma

Ghasaq Al Obaidi[1], Yusuf Saleeby[2], Joseph Varon[3], Natalie Durzynski[4], Jack Tuszynki[5,6] and Matthew Halma[4,*]

[1]*Internal Medicine, Baghdad Medical City, University of Baghdad, Baghdad, Iraq*
[2]*Carolina Holistic Medicine, Mount Pleasant, SC, USA*
[3]*Independent Medical Alliance, Washington, D.C., USA and Dorrington Medical Associates Internal Medicine/Pulmonology, Houston, Texas, USA*
[4]*Open Source Medicine Foundation, Tallinn, Estonia*
[5]*Unviersity of Alberta, Edmonton, Alberta, Canada*
[6]*Politecnico di Torino, Torino, Italy*

© 2026 The Author(s). Published by Bentham Open.
This is an open access article distributed under the terms of the Creative Commons Attribution 4.0 International Public License (CC-BY 4.0), a copy of which is available at: https://creativecommons.org/licenses/by/4.0/legalcode. This license permits unrestricted use, distribution, and reproduction in any medium, provided the original author and source are credited.

Received: November 10, 2025
Revised: January 09, 2026
Accepted: January 21, 2026
Published: May 14, 2026

*Address correspondence to this author at the Open Source Medicine Foundation, Tallinn, Estonia;
E-mail: mhalma@imahealth.org

Cite as: Al Obaidi G, Saleeby Y, Varon J, Durzynski N, Tuszynki J, Halma M. Beyond Conventional Treatment: Herbal Medicine and Nutraceuticals as Complementary Therapies for COPD and Asthma. Open Respir Med J, 2026; 20: e18743064454037. http://dx.doi.org/10.2174/0118743064454037260510201039

Send Orders for Reprints to reprints@benthamscience.net

Table S1. An overview of the therapeutic agents included in articles on herbal treatment for COPD and asthma.

General Review	Therapeutics
(Cao *et al*. 2023) [3]	Artesunate, Cimigenoside, Astragaloside, Andrographolide, Crocin, Celastrol, Betulin, Eucalyptus oil, Baicalin, Naringin, Icariin, Total flavonoids, Quercetogetin, Quercetin, Houttuynin, Curcumin, Resveratrol, Chrysophanol, Acetylshikonin, Magnolia fargesii, Sodium tanshinone IIA sulfonate, Salidroside, Ophiocordyceps sinensis, Stemonine, Matrine
(Isago *et al*. 2021) [8]	Astragali radix, Atractyloclis lancea rhizoma, Ginseng radix, Angelicae radix, Bupleuri radix, Zizyphi fructus, Aurantii nobilis pericarpium, Glycyrrhizae radix, Cimicifugae rhizoma, Zingiberis rhizome. These components are part of the Japanese herbal Kampo medicine known as Hochuekkito (TJ-41)
(Sahin and Sahin 2013) [20]	Mallow tea, Nigella, Honey, Mint tea, Sage tea, Rosehip, Thyme tea, Lemon balm tea, Green tea
(Lin *et al*. 2019) [13]	Fritillaria thunbergii, Prunus armeniaca, Platycodon grandiflorus, Scutellaria baicalensis, Ophiopogon japonicus, Houttuynia cordata, Salvia miltiorrhiza, Glycyrrhiza uralensis, Tussilago farfara, Magnolia officinalis
(Lyu *et al*. 2022) [14]	Ginseng, Glycyrrhiza, Schisandra, Sophora, Astragalus, Angelica, Polygonatum, Lycium
(Rahminiwati *et al*. 2022) [18]	Adenostemma lavenia, Apium graveolens (*Celery*), Centella asiatica, Cosmos caudatus, Muntingia calabura, Zingiber officinale (*Red Ginger*)
(Poursaleh *et al*. 2022) [60]	Ginger, Cinnamon, Saffron, Cardamom, Galangal. The above are ingredients of compound honey syrup.
(Baniamerian *et al*. 2023) [2]	Zataria multiflora, Thymus vulgaris, Glycyrrhiza glabra, Crocus sativus, Nepeta bracteata, Hyssopus officinalis
(Panda *et al*. 2023) [15]	Curcuma longa (*Turmeric*), Zingiber officinale (*Ginger*), Panax ginseng, Astragalus membranaceus, Cordyceps sinensis, Glycyrrhiza glabra (*Licorice*), Ephedra sinica, Radix platycodonis, Morus alba, Scutellaria baicalensis, Coptis chinensis

(Table) contd.....

General Review	Therapeutics
(Ram *et al*. 2011) [1]	*Acacia farnesiana, Acacia leucophloea, Acacia nilotica, Acacia torta, Acalypha indica, Adhatoda vasica, Ailanthus excelsa, Ailanthus malabarica, Althaea officinalis, Allium cepa, Amorphophallus campanulatus, Apium* graveolens, Atropa acuminate, Brassica nigra, Boswellia serrata, Calotropis gigantean, Castaneae folium, Cassia sophera, Chelidonium majus, Cimicifuga racemosa, Clitoria ternatea, Coleus amboinicus, Convolvulus pluricaulis, Datura stramonium, Dorema ammoniacum, Echinacea purpurea, Elittaria cardamomum, Emblica officinalis, Ephedra sinica, Eugenia caryophyllata, Euphorbia hirta, Foeniculum vulgare, Glycyrrhiza glabra, Gonoderma lucidum, Hederae helix, Inula helenium, Iris germanica, Lactuca virosa, Laretia acaulis, Malva sylvestris, Matricaria recutita, Melilotus indica, Mentha heplocalyx, Ocimum sanctum, Parthenium integritolium, Prunus armenica, Pimpinella anisum, Piper cubeba, Piper longum, Plantago ovata, Polygala sibirica, Serenoa repens, Seutellaria baicaleneis, Sida cordifolia, Solanum trilobatum, Solanum xanthocarpum, Symphytum officinale, Taxus baccata, Trachyspermum ammi, Trifolium pretense, Turnera aphrodisiaca, Tussilago farfara, Ulmus fulva, Verbena officinalis, Viola tricholor, Withania somnifera, Yucca gloriosa, Zanthoxylum budrunga, Zingiber officinale, Zanthoxylum oxyphyllum
(Santana *et al*. 2016) [21]	Eriodictyol, Luteolin, Quercetin, Kaempferol, Mitraphylline, Asperuloside, Eugenol, Sakuranetin, Kuwanon G, Naringin, Apigenin, Extracts from *Astragalus membranaceus, Boerhavia procumbens, Ocimum gratissimum, Punica granatum, Siegesbeckia glabrescens, Callicarpa japonica,* and others, Several polyphenols from green tea (epicatechin, epigallocatechin, epicatechin gallate, epigallocatechin gallate), Resveratrol
(Zhen, *et al*. 2018) [25]	Xiaoqinglong decoction (XQLD): *Ephedrae Herba* (Ma Huang), *Asari Radix et Rhizoma* (Xi Xin), *Glycyrrhizae Radix et Rhizoma* (Gan Cao), *Zingiberis Rhizoma* (Gan Jiang), *Pinelliae Rhizoma* (Ban Xia), *Schisandrae Chinensis Fructus* (Wu Wei Zi), *Paeoniae Radix Alba* (Bai Shao), *Ramulus Cinnamomi* (Gui Zhi)
(Willcox *et al*. 2023) [22]	Shufeng Jiedu (SFJD): *Reynoutria japonica* (Japanese Knotweed), *Forsythia suspensa* (Weeping Forsythia), *Isatis tinctoria* (Indigo Woad), *Bupleurum chinense* (Chinese Thoroughwax), *Patrinia scabiosifolia* (Yellow Flowered Valerian), *Verbena officinalis* (Vervain), *Phragmites australis* (Common Reed), Glycyrrhiza uralensis (Chinese Liquorice)
(Zhang *et al*. 2019) [24]	Yihuo Huatan Formula (YHF): Quercetin, Kaempferol, Luteolin, Stigmasterol, Baicalein, 7-O-methylisomucronulatol, Ellagic acid
(Chen *et al*. 2014) [25]	*Astragalus membranaceus*, Panax ginseng, *Cordyceps sinensis*
(An *et al*. 2012) [1]	Huangqi, Baizhu, Dangshen, Fuling, Wuweizi, *Astragalus membranaceus*, *Atractylodes macrocephala*, *Codonopsis pilosula*, *Poria cocos, Schisandra chinensis*
(Li *et al*. 2021) [10]	20-S-ginsenoside Rh1, astragaloside IV, icariin, nobiletin, paeonol
(Li *et al*. 2021) [12]	20-S-ginsenoside Rh1, astragaloside IV, icariin, nobiletin, paeonol
(Xiong *et al*. 2021) [23]	Buzhongyiqi granule (renshen, huangqi, baizhu, danggui, chenpi, fangfeng, shenma, fuling, yuzhu, shashen), Naqitongluo decoction (huangqi, shanzhuyu, buguzhi, chenxiang, ganjiang, xixin, wuweizi, jiegeng, tanglizi, taoren, chuanxiong, houpo, chenpi), Yiqijianpi granules (dangshen, baizhu, fuling, huaishanyao, chenpi, shugeng, zhigancao), Sijunzitang and Jinkuishenqiwan decoction (rensheng, fuling, baishu, gancao, guizhi, shudihuang, shanzhuyu, shanyao, danpi, zhexie, zhifupian), Yiqigubiao pill (dang shen, fuxiaomai, baizhu, banxia, chenpi, zisu, fuling, fangfeng, yiyiren, kuandonghua, huangqin, chuanbeimu, pipaye), Suzijiangqitang granule (suzi, banxia, danggui, qianhu, houpo, rougui, suye, zhigancao), Yufeining granule (dangshen, huangqi, baishu, fangfeng, huangjing, shanzhuyu, wuweizi, hetaorou, tusizi, bajitian, gualou, fabanxia, zhebeimu, dangshen, taoren), Yupingfeng granules (huangqi, baizhu, fangfeng), Jianpibushenhuatan decoction (dangshen, huanqgi, zishuzi, zishiying, maozhucao, fuling, zhibanxia, chenpi, baizhu, taoren, xinren, shudi, dilong), Effective compound combination (ECC; 20-S-ginsenoside Rh1, astragaloside, icariin, nobiletin, paeonol)
(Possebon *et al*. 2018) [16]	*Arctium lappa, Plantago major, Mikania glomerata Spreng, Equisetum arvense*
(Chen *et al*. 2014) [4]	*Arctium lappa, Plantago major, Mikania glomerata Spreng, Equisetum arvense*
(Kwon *et al*. 2021) [9]	*Poria cocos, Citrus unshiu, Codonopsis pilosulae, Atractylodes macrocephala, Glycyrrhiza uralensis, Cornus officinalis, Rehmannia glutinosa, Astragalus membranaceus, Paeonia lactiflora, Prunus persica, Rheum palmatum, Prunus armeniaca, Pinellia ternata, Carthamus tinctorius, Pheretima aspergillum, Sinapis alba, Raphanus sativus*
(Clarke, Lundy, and McGarvey 2015) [6]	*Atropa belladonna, Ganoderma lucidem, Sophora flavescentis, Glycyrrhiza uralensis, Aleurites moluccana, Nigella sativa, Magnoliae Flos, Echinacea purpurea, Epimedium, Chinese yam, Tanreqing*
(Li *et al*. 2020) [11]	Ephedra (*Ephedrae herba*), Cassia twig (*Cinnamomi ramulus*), Dried ginger (*Zingiberis rhizoma*), Debark peony root (*Paeoniae Radix Alba*), Processed pinellia tuber (*Pinelliae Rhizoma Praeparatum*), Chinese magnoliavine fruit (*Schisandrae Chinensis Fructus*), Manchurian wildginger (*Asari Radix Et Rhizoma*), Perilla fruit (*Perillae fructus*), Bitter Apricot Seed (*Armeniacae Semen Amarum*), Officinal magnolia bark (*Magnoliae Officinalis Cortex*), Prepared Radix Glycyrrhizae (*Glycyrrhizae Radix Et Rhizoma Praeparata Cum Melle*), Snakegourd fruit (*Trichosanthis fructus*), Alum processed pinellia (*Pinelliae Rhizoma Praeparatum Cum Alumine*), Thunberg fritillary bulb (*Fritillariae Thunbergii Bulbus*), Cape jasmine fruit (*Gardeniae fructus*), White mulberry root-bark (*Mori Cortex*), Baical skullcap root (*Scutellariae Radix*), Chinese pulsatilla root (*Pulsatillae Radix*), Heartleaf houttuynia herb (*Houttuyniae Herba*), Peony root (*Paeoniae Radix Rubra*), American ginseng (*Panacis Quinquefolii Radix*), Dwarf lilyturf tuber (*Ophiopogonis Radix*), Dried tangerine peel (*Citri Reticulatae Pericarpium*), Longstamen onion bulb (Allii Macrostemonis Bulbus), Indian bread (*Poria*), Pepperweed seed (*Descurainiae Semenlepidii Semen*), Mustard seed (*Sinapis Semen*), Ginseng (*Ginseng Radix Et Rhizoma*).
(Guo, *et al*. 2006) [7]	Panax ginseng, Salvia miltiorrhiza, Jinshui Liujin decoction, Jiawei Yupingfeng decoction, Echinacea, Hedera helix, Ke Chuan Ping decoction, 13-Herb anti-cough–dyspnoea decoction, Yiqi Mianyi granule, Prospan cough syrup, Prospan herbal drops, Kesuning granule, Bufei Keli granule, Gubenhuatanquyu decoction

REFERENCES

[1] An X, Zhang AL, May BH, Lin L, Xu Y, Xue CC. Oral Chinese herbal medicine for improvement of quality of life in patients with stable chronic obstructive pulmonary disease: a systematic review. J Altern Complement Med 2012; 18(8): 731-43. http://dx.doi.org/10.1089/acm.2011.0389 PMID: 22803654

[2] Baniamerian R, Tahermohammadi H, Daneshfard B, *et al.* Herbal treatment of COPD and Asthma According to Persian medicine: A review of current evidence. Tanaffos 2023; 22(2): 187-99. PMID: 38628881

[3] Cao X, Wang Y, Chen Y, *et al.* Advances in traditional Chinese medicine for the treatment of chronic obstructive pulmonary disease. J Ethnopharmacol 2023; 307116229 http://dx.doi.org/10.1016/j.jep.2023.116229 PMID: 36773789

[4] Chen HY, Ma CH, Cao KJ, *et al.* A systematic review and meta-analysis of herbal medicine on chronic obstructive pulmonary diseases. Evid Based Complement Alternat Med 2014;

2014(1)925069
http://dx.doi.org/10.1155/2014/925069 PMID: 24795773

[5] Chen X, May B, Di YM, *et al.* Oral Chinese herbal medicine combined with pharmacotherapy for stable COPD: a systematic review of effect on BODE index and six minute walk test. PLoS One 2014; 9(3)e91830
http://dx.doi.org/10.1371/journal.pone.0091830 PMID: 24622390

[6] Clarke R, Lundy FT, McGarvey L. Herbal treatment in asthma and COPD – current evidence. Clinical Phytoscience 2015; 1(1): 4.
http://dx.doi.org/10.1186/s40816-015-0005-0

[7] Guo R, Pittler MH, Ernst E. Herbal medicines for the treatment of COPD: a systematic review. Eur Respir J 2006; 28(2): 330-8.
http://dx.doi.org/10.1183/09031936.06.00119905 PMID: 16880367

[8] Isago H, Mitani A, Kohno S, *et al.* The Japanese herbal (Kampo) medicine Hochuekkito attenuates lung inflammation in lung emphysema. Biol Pharm Bull 2021; 44(1): 39-45.
http://dx.doi.org/10.1248/bpb.b20-00556 PMID: 33390548

[9] Kwon CY, Lee B, Lee BJ, Kim KI, Jung HJ. Herbal medicine compared to placebo for chronic obstructive pulmonary disease: A systematic review and meta-analysis. Front Pharmacol 2021; 12717570
http://dx.doi.org/10.3389/fphar.2021.717570 PMID: 34744711

[10] Li J, Xie Y, Zhao P, *et al.* A chinese herbal formula ameliorates COPD by inhibiting the inflammatory response *via* downregulation of p65, JNK, and p38. Phytomedicine 2021; 83153475
http://dx.doi.org/10.1016/j.phymed.2021.153475 PMID: 33545548

[11] Li J, Zhang H, Ruan H, *et al.* Effects of Chinese herbal medicine on acute exacerbations of COPD: A randomized, placebo-controlled study. Int J Chron Obstruct Pulmon Dis 2020; 15: 2901-12.
http://dx.doi.org/10.2147/COPD.S276082 PMID: 33209019

[12] Li J, Zhao P, Tian Y, *et al.* The anti-inflammatory effect of a combination of five compounds from five Chinese herbal medicines used in the treatment of COPD. Front Pharmacol 2021; 12709702
http://dx.doi.org/10.3389/fphar.2021.709702 PMID: 34776947

[13] Lin TH, Chen SI, Su YC, Lin MC, Lin HJ, Huang ST. Conventional Western treatment combined with Chinese herbal medicine alleviates the progressive risk of lung cancer in patients with chronic obstructive pulmonary disease: A nationwide retrospective cohort study. Front Pharmacol 2019; 10: 987.
http://dx.doi.org/10.3389/fphar.2019.00987 PMID: 31572178

[14] Lyu YR, Lee SW, Kim SY, *et al.* Herbal medicines for the treatment of chronic obstructive airway diseases (Asthma or Chronic Obstructive Pulmonary Disease): A prospective observational study. Evid Based Complement Alternat Med 2022; 2022: 1-12.
http://dx.doi.org/10.1155/2022/3485757 PMID: 35677382

[15] Panda SR, Jain S, Syamprasad NP, *et al.* Herbal medicines for the treatment of COPD.Role of Herbal Medicines. Singapore: Springer 2023; pp. 167-84.
http://dx.doi.org/10.1007/978-981-99-7703-1_9

[16] Possebon L, de Souza Lima Lebron I, Furlan da Silva L, *et al.* Anti-inflammatory actions of herbal medicines in a model of chronic obstructive pulmonary disease induced by cigarette smoke. Biomed Pharmacother 2018; 99: 591-7.
http://dx.doi.org/10.1016/j.biopha.2018.01.106 PMID: 29710457

[17] Poursaleh Z, Choopani R, Vahedi E, *et al.* Effect of herbal medicine formulation (Compound Honey Syrup) on quality of life in patients with COPD: A randomized clinical trial. Tanaffos 2022; 21(3): 336-47.
PMID: 37025308

[18] Rahminiwati M, Trivadila T, Iswantini D, *et al.* Indonesian medicinal plants with anti-inflammatory properties and potency as chronic obstructive pulmonary disease (COPD) herbal medicine. Pharmacogn J 2022; 14(4): 432-44.
http://dx.doi.org/10.5530/pj.2022.14.119

[19] Ram A, Balachandar S, Vijayananth P, Singh VP. Medicinal plants useful for treating chronic obstructive pulmonary disease (COPD): Current status and future perspectives. Fitoterapia 2011; 82(2): 141-51.
http://dx.doi.org/10.1016/j.fitote.2010.09.005 PMID: 20851752

[20] Şahin ZA, Şahin M. The view of patients with Chronic Obstructive Pulmonary Disease (COPD) on complementary and alternative medicine (CAM) in eastern Turkey. Afr J Tradit Complement Altern Med 2013; 10(4): 116-21.
http://dx.doi.org/10.4314/ajtcam.v10i4.19 PMID: 24146511

[21] Santana FPR, Pinheiro NM, Mernak MIB, *et al.* Evidences of herbal medicine-derived natural products effects in inflammatory lung diseases. Mediators Inflamm 2016; 2016: 1-14.
http://dx.doi.org/10.1155/2016/2348968 PMID: 27445433

[22] Willcox ML, Hu XY, Oliver T, *et al.* Treating acute exacerbations of COPD with Chinese herbal medicine to aid antibiotic use reduction (Excalibur): a randomised double-blind, placebo-controlled feasibility trial. Front Pharmacol 2023; 141221905
http://dx.doi.org/10.3389/fphar.2023.1221905 PMID: 37818189

[23] Xiong C, Li Y, Zhuang G, *et al.* Clinical efficacy and safety of Chinese herbal medicine *versus* placebo for the treatment of chronic obstructive pulmonary disease: A systematic review and meta-analysis. Complement Ther Med 2021; 59102691
http://dx.doi.org/10.1016/j.ctim.2021.102691 PMID: 33618010

[24] Zhang XW, Liu W, Jiang HL, Mao B. Dissection of pharmacological mechanism of Chinese herbal medicine Yihuo Huatan formula on chronic obstructive pulmonary disease: A systems pharmacology-based study. Sci Rep 2019; 9(1): 13431.
http://dx.doi.org/10.1038/s41598-019-50064-9 PMID: 31530860

[25] Zhen G, Jing J, Fengsen L. Traditional Chinese medicine classic herbal formula Xiaoqinglong decoction for acute exacerbation of chronic obstructive pulmonary disease. Medicine (Baltimore) 2018; 97(52)e13761
http://dx.doi.org/10.1097/MD.0000000000013761 PMID: 30593152
